# Dyspnea as the Reason for Encounter in General Practice

**DOI:** 10.4021/jocmr642w

**Published:** 2011-09-26

**Authors:** Thomas Frese, Caroline Sobeck, Kristin Herrmann, Hagen Sandholzer

**Affiliations:** a Department of Primary Care, Leipzig Medical School, Leipzig, Germany; c These authors contributed equally to the recent work.

## Abstract

**Background:**

Dyspnea is a common reason for consulting a physician. Data from the primary care setting on the epidemiology, management, and underlying causes of dyspnea have seldomly been published. The present study is aimed to explore the consultation prevalence of dyspnea, frequency of diagnostic and therapeutic procedures, accompanying symptoms and results of encounter or diagnoses of patients with dyspnea in a day-to-day primary care setting.

**Methods:**

Cross-sectional data were collected from randomly selected patients during the SESAM 2 study (October 1, 1999 to September 30, 2000). Unpublished but publicly available data from the Dutch Transition Project were also analysed.

**Results:**

One (n = 93; SESAM 2) and 3.9% (n = 7,855; Transition Project) of the patients consulted the practioner for dyspnea. The male to female ratio was almost 1 : 1. Half of the patients sought medical advice for not previously known dyspnea (Transition Project). Dyspnea occurs more frequently among small children (0 to 4 years) and elderly adults (> 64 years of age). Nearly all patients received a physical examination. Many causes were examined with the help of electrocardiograms but spirometry and laboratory tests were also used. Drug prescription was the most frequent (79.6%) therapeutic procedure. Acute bronchitis was the most common diagnosis. Dyspnea was significantly associated to cough, dysphagia, abnormal sputum, airway pain, sweating, and thoracic pain. There was also a significant association to chronic obstructive pulmonary disease.

**Conclusions:**

Dyspnea is a common reason for seeking medical advice. Emergency cases (e.g. myocardial infarction) are rarely present in the general practitioner’s consultation. The majority of underlying causes are respiratory tract infections and exacerbated, previously known chronic diseases.

**Keywords:**

Dyspnea; General practice; Primary care; Reason for encounter

## Introduction

A patient presents to a general practitioner with dyspnea. Is this a simple case that can be handled in the doctor’s office or could the cause be myocardial infarction, a life-threatening asthmatic attack or pulmonary embolism requiring urgent hospital treatment? For the sensation of dyspnea there is no clear, established definition because the affected patients have different afflictions [1,2]. Dyspnea is an unignorable ”air hunger“ [2-4].

Dyspnea is a common symptom in general practice [1,3,5,6]. It occurs as one of the reasons for consulting in 4% of all consultations [5]. Acute dyspnea is one of the most common paediatric emergencies [7] but it occurs more often with increasing age [8]. It is distressing [1,5], and may be caused by many different disorders [1]. The underlying causes range from simple cases handled with outpatient treatment to very serious and life-threatening emergencies requiring urgent clarification [1].

The present study was planned to characterize the consultation prevalence, the management, the results of encounter or the differential diagnoses, and the significantly more frequent co-morbidities of patients attending a typical primary care setting. The registration of the contacts for dyspnea was a minor part of the study. Unpublished data from the Dutch Transition Project – another primary care study – were also analyzed with regard to dyspnea as the reason for the consultation.

## Materials and Methods

The Saxon Society of General Medicine (SGAM) contacted all general practitioners in Saxony by mail. They received no incentive for the participation. The study was set out to document reasons for consultation, diagnostic, and therapeutic procedures as well as the result of consultation (chosen diagnosis).

Of the 2,510 physicians contacted, 270 general practitioners agreed to participate and 209 cooperated during the complete period of the study (one year). Cross-sectional data were collected from October 1, 1999 to September 30, 2000. Case recording was carried out on one day a week (Monday to Friday; either morning or afternoon consultation hours), chosen at random. Data were collected for one out of ten patients of every practitioner (exactly every tenth patient attending the consultation hour). Multiple recording of the same patient was avoided. House calls were not considered. A total of 8,877 patients were included.

A standardized paper-based data collection form was used [9]. It was developed by general practitioners (Leipzig Medical School and Saxon Society of General Medicine). The form was tested and evaluated during a pilot trial (Saxon Epidemiological Study in General Practice - SESAM 1). Each patient’s reasons for consulting, symptoms, diagnostic procedures, recent diagnoses, and general morbidity were documented as well as therapeutic procedures. As far as possible, data were documented verbatim (according to the study instructions): either as told by the patients (e.g. reasons for consultation) or in the words of the physician (e.g. chronic problems, disease labels, or diagnoses). Only completely filled-in forms were considered.

As described elsewhere, the SESAM 2 study provides independent and unbiased cross-sectional data from a typical primary care setting [10,11]. Because all reasons for consulting were investigated and documented there is no bias towards the investigated reason for consulting. The 1987 version of the International Classification of Primary Care (ICPC) was used to code the reasons for the consultation [12]. The SESAM 2 data were compared to those of two other studies. Unpublished, but publicly available data from the Dutch Transition Project (described by Lamberts and Okkes [13]) were analyzed (total estimation of patients from about 20 Dutch general practitioners; 1985 till 2003). The data are available at www.transitieproject.nl. It can be analysed using the software the database provides.

The performance of the SESAM 2 study was in accordance to the guidelines of the institutional review board/ethics committee. As stated by the ethics committee no special approval was demanded.

Statistical analyses of the data were performed using Statistical Packages for Social Sciences (SPSS 15.0; SPSS Inc., Chicago, USA). As indicated, data were compared using Fisher’s exact test. Differences were stated as statistically significant for p < 0.05.

## Results

### SESAM-Study

A total of 8,877 consultations were documented in the SESAM 2 study. 13,632 reasons for consultation were coded. The number of cases reported from each doctor’s surgery ranged from 23 to 54. 5,050 (56.9%) female and 3,824 (43.1%) male patients were reported by 209 general practitioners; gender was not reported in 3 cases. Age ranged from 2 to 102 years (mean 51.2 years, SD ± 20.86, median 55 years). Of all the patients, 93 (1.05%) attended for dyspnea. The consultation prevalence (prevalence of dyspnoeic patients in general practitioners consultation) was higher in children (0 - 14 years) than in adults. The highest consultation prevalence of 1.7% was seen in patients older than 75 years (Table 1). The consultation prevalence was slightly lower in men (0.9%) than in women (1.1%).

**Table 1:** Patient Distribution* (pd) on Different Age Groups and Consultation Prevalence** (cp) of Dyspnea in Different Age Groups of the German SESAM 2 Study and the Dutch Transition Project Concerning the Condition of New or Previously Known Dyspnea in a General Practice Setting.

**Age (years)**	**SESAM 2 study (n = 93)**		**Transition Project initial consultation for new occurred dyspnea (n = 3743)**		**Transition Project subsequent consultation for previously known dyspnea (n = 2747)**	
	**pd (%)**	**cp (%)**	**pd (%)**	**cp (%)**	**pd (%)**	**cp (%)**
0 to 4	1.08	0.90	12.49	4.8	6.64	2.2
5 to 14	3.23	1.06	7.15	1.9	6.98	1.5
15 to 24	6.45	0.68	7.59	1.8	5.74	1.1
25 to 44	15.05	0.77	24.05	2.0	18.36	1.2
45 to 64	26.88	0.87	22.62	2.5	24.08	1.9
65 to 74	24.73	1.44	13.7	3.2	18.99	3.0
>75	21.51	1.74	12.42	3.1	19.20	3.4

*Patient distribution (pd) is the percentage of the specified age group of the patients with dyspnea (e.g. 3.23 % of all SESAM 2-patients with dyspnea were 5 to 14 years old); **Consultation prevalence (cp) is the percentage of patients with dyspnea in the age group related to all patients of the age group (e.g. among SESAM 2-children from 5 to 14 years of age 1.06% encounter for dyspnea).**There were no patients under age 2 who presented with dyspnea in the SESAM 2 study.

Some accompanying symptoms were significantly associated with dyspnea: patients encountering for dyspnea in the SESAM 2 study suffered significantly more often from cough, dysphagia, abnormal sputum, airway pain, and sweating (p < 0.01 for each) than those patients without dyspnea. Other associated symptoms were musculoskeletal chest pain (p = 0.019) and angina pectoris (p = 0.037).

Nearly all patients received a physical examination (93.5%). The implemented diagnostic procedures included electrocardiogram (ECG, 26.9%), spirometry (18.3%), and laboratory tests (16.1%). Further tests were rarely performed (Table 2). The majority (84.9%) of the patients had one more appointment.

**Table 2:** Physician’s Action (%) in the SESAM 2 Study and the Transition Project Concerning the Condition of New or Already Known Dyspnea. Procedures That Are Not Explicit Diagnostic Were Also Registered in Table 3

**Physician's action**	**SESAM 2 study (n = 93)**	**Transition Project initial consultation for new occurred dyspnea (n = 3743)**	**Transition Project subsequent consultation for previously knowndyspnea (n = 2747)**
Physical examination	93.55	99.48	92.36
Follow-up consultation	84.95	N/A	N/A
EKG	26.88	N/A	N/A
Spirometry	18.28	N/A	N/A
Laboratory investigations	16.13	6.36	5.11
Other diagnostics	13.98	0.27	0.90
Referral	8.60	1.50 (p.c.)*	1.79 (p.c.)*
Anamnesis	4.30	N/A	N/A
Hospitalisation	1.08	3.62 (s.c.)**	5.40 (s.c.)**

*pc: primary care; **sc: specialized care.

The most frequent therapeutic procedure was drug prescription (79.6%). 11.8% received individual consultancy as well as a disability certificate. Referral to hospital was necessary in only one case (Table 3). Most (84.9%) of the results of consultation belonged to the International Classification of Diseases-chapters respiratory tract (69.9%) or cardiovascular system (15%). The most frequent consultation result was acute bronchitis, followed by heart failure, chronic obstructive pulmonary disease (COPD), asthma, chronic bronchitis, pneumonia, and dyspnea itself. Table 4 lists the incidence of new diagnoses with significant association to dyspnea in the study patients. The potentially dangerous causes myocardial infarction, pneumonia, and cardiac arrhythmia were rarely diagnosed. Table 5 summarizes the consultation prevalence of the most frequent final diagnoses in patients with dyspnea.

**Table 3:** Physician’s Action (%) in the SESAM 2 Study and the Transition Project Concerning the Condition of New or Already Known Dyspnea. Procedures That Are Not Explicitly Therapeutic Were Also Registered in Table 2

**Physician's action**	**SESAM 2 study (n = 93)**	**Transition Project initial consultation for new occurred dyspnea (n = 3743)**	**Transition Project subsequent consultation for previously knowndyspnea (n = 2747)**
Follow-up consultation	84.95	N/A	N/A
Drug prescription	79.57	58.73	63.59
Other therapy	12.90	0.05	0.08
Incapacity to work	11.83	N/A	N/A
Physicians advice	11.83	30.52	28.32
Referral	8.60	1.50 (p.c.)*	1.79 (p.c.)*
Physiotherapy	5.38	N/A	N/A
Long-term care new	4.30	N/A	N/A
Vaccination	2.15	N/A	N/A
Hospitalisation	1.08	3.62 (s.c.)**	5.40 (s.c.)**

*pc: primary care; **sc: specialized care.

**Table 4:** Comparing the Incidence (Number (N) and Percentage (%)) of Results of Encounter (“Diagnoses”) in General Practice Patients With Dyspnea to Those Without Dyspnea (SESAM 2 Study) Shows That Dyspnea Is Significantly Associated to Cardiopulmonary Diseases

Diagnosis*	**Dyspnea (n = 93)**		**Without dyspnea (n = 8784)**		**p-Value**
		
	absolute	(%)	absolute	(%)	(Fisher)
Acute bronchitis/bronchiolitis	23	24.73	296	3.37	0.000
Other airway diseases	6	6.45	14	0.16	0.000
Heart failure	5	5.38	26	0.30	0.000
COPD	5	5.38	19	0.22	0.000
Bronchial asthma	5	5.38	19	0.22	0.000
Chronic bronchitis	4	4.30	16	0.18	0.000
Dyspnea	4	4.30	2	0.02	0.000
Pneumonia	3	3.23	26	0.30	0.003
Other respiratory infection	1	1.08	3	0.03	0.041
Complaints related to respiratory system organs	1	1.08	2	0.02	0.031

*Further diagnoses of patients with dyspnea omitted from the table because of lacking significance: acute upper respiratory infection, health maintenance/preventive medicine, oedema, ischaemic heart disease with angina, acute myocardial infarction, atrial fibrillation/flutter, cardiac arrhythmia (not other specified), uncomplicated hypertension, low back symptom/complaint, other osteoarthrosis, sleep disturbance, somatisation disorder, other breathing problem, acute/chronic sinusitis, acute laryngitis/tracheitis, influenza and obesity/overweight.

**Table 5:** Incidence (%) of the Most Frequent Diagnoses for Primary Care Patients With Complaints of Dyspnea.

**Diagnosis**	**SESAM 2 study (n = 93)**	**Transition Project initial consultation for new occurred dyspnea (n = 3743)**	**Transition Project subsequent consultation for previously known dyspnea (n = 2747)**
Acute bronchitis	24.73	24.51	9.60
Acute upper respiratory infection	9.68	6.90	0.87
Other airway infection	6.45	0.10	0.05
Bronchial asthma	5.38	10.96	36.12
COPD	5.38	2.19	15.86
Heart failure	5.38	4.09	6.98
Chronic bronchitis	4.30	0.79	5.11
Essential hypertension	4.30	0.25	0.55
Acute shortness of breath/dyspnea	4.30	14.12	4.79
Pneumonia	3.23	2.64	1.03
Acute laryngitis/tracheitis	2.15	3.92	1.00
Prevention/no disease	2.15	1.50	0.16
Ischemic heart disease	2.15	1.23	1.66
Obesity	1.08	0.12	0.08
Back pain	1.08	0.07	0.03
Acute myocardial infarction	1.08	0	0
Acute/chronic sinusitis	1.08	1.78	0.32
Atrial fibrillation/flutter	1.08	0.49	0.79
Influenza	1.08	0.44	0.03
Cardiac arrhythmia	1.08	0.15	0
Oedemata	1.08	0	0
Osteoarthritis	1.08	0	0
Sleep disorder	1.08	0	0
Allergic rhinitis	0	0.57	0.95
Depressive episode	0	0.12	0.21
Pulmonary embolism	0	0.35	0.21
Hyperventilation	0	7.56	4.01
Cough	0	2.05	0.84
Acute stress	0	0.44	0.18
Acute tonsillitis	0	0.35	0.18
Iron deficiency anaemia	0	0.20	0.08
Anxiety disorder	0	0.15	0.37
Malignant respiratory system neoplasm	0	0.12	0.40
Fear of heart attack	0	0.07	0
Stroke	0	0	0.05

*Further diagnoses of patients with dyspnea omitted from the table because of lacking significance: acute upper respiratory infection, health maintenance/preventive medicine, oedema, ischaemic heart disease with angina, acute myocardial infarction, atrial fibrillation/flutter, cardiac arrhythmia (not other specified), uncomplicated hypertension, low back symptom/complaint, other osteoarthrosis, sleep disturbance, somatisation disorder, other breathing problem, acute/chronic sinusitis, acute laryngitis/tracheitis, influenza and obesity/overweight.

### Transition Project

149,238 patients were examined in the Transition Project over the period 1985 - 2003. 84,285 (56.5%) of them were female. About four percent (6,490 patients) declared dyspnea as the reason for consultation. 3743 (57.7%) of these attended for the first time with dyspnea, 2,747 had previously known dyspnea. The age distribution of the patients is given in Table 1.

Patients presenting new dyspnea often also suffered from cough (23.3%), fever (3%), and general weakness / tiredness (2.9%). With new dyspnea were also associated upper respiratory infection (URI, 1.9%), chest symptoms / complaints (1.7%), wheezing (1.6%), and pain attributed to the respiratory system (1.1%).

Most patients received a physical examination (Table 2). Medication was prescribed or injections were given in 58.7% of the patients presenting new dyspnea and in 63.6% of the patients with known dyspnea. Health education or medical advice was given in about 30% of the cases in both groups. Further diagnostic investigation was not necessary in most of the cases (Table 2). A referral to other specialised physicians or a hospital was made in 3.6% (new dyspnea) to 5.7% (known dyspnea; Table 3) of the cases. In both groups most patients were sent to a respiratory or cardiologic physician. The most frequent emergency referral was to an emergency respiratory physician.

The results of encounter are listed in Table 5: Dyspnea was caused by infections of the airways, chronic cardiac or respiratory diseases. Myocardial infarction or pulmonary embolism was rare.

## Discussion

Dyspnea is a common complaint a general practitioner has to deal with [1,3,5,6]. Data from a primary care setting have rarely been published [14]. The consultation prevalence of new occurrences of dyspnea in the studies varied from about 1.0% in the SESAM 2 study to 2.5% in the Transition Project. Middle-aged men suffered more frequently from dyspnea than other men. This may be explained by the higher tobacco consumption than compared to other age groups or women [15,16]. Huijnen did not report age-related prevalence changes of dyspnea in men while older women suffered more often from dyspnea than younger ones [17]. Currow et al. found that dyspnea is significantly associated with female gender and higher age [18]. According to this the SESAM 2 study and the Transition Project indicated that the consultation prevalence of dyspnea increased with age (Table 1). This might be due to the fact that chronic cardiac or pulmonary diseases occur more frequently among the elderly [1]. Related to a different consultation behaviour (children in Germany are usually treated by paediatricians) in the Transition Project the highest consultation prevalence of dyspnea was found in children from 0 to 4 years of age (Table 1). This can be explained by the high frequency of upper respiratory tract infections and cough in this age group [19]. As confirmed by the recent data both problems may be accompanied by dyspnea. We found that dyspnea was associated to other reasons for consulting in two-thirds of the cases. Our results concur with earlier reported findings from an Australian primary care setting [8]. Charles et al. reported cough (16.9%), chest pain (5%), fatigue (3.5%), request for medication (3.2%), cardiovascular screenings (2.5%), wheezing (2.4%), and swollen ankles or oedema (1.8%) as accompanying reasons for consultations of dyspnoeic patients [8].

In the SESAM 2 study and the Transition Project, nearly all patients presenting with dyspnea received a physical examination or health evaluation. In contrast to other problems, a basic diagnostic program including electrocardiogram, spirometry and laboratory investigations is helpful and was reported to be usually performed in dyspnoeic patients [1,14,15,20,21]. An electrocardiogram or spirometry was performed in 26.9% or 18.3% of the dyspnoeic patients in the SESAM 2 study. These rates appear low. But as indicated by the most frequent results of encounter or diagnoses (Table 5) it becomes clear that the underlying cause of dyspnea may not be elucidated by the electrocardiogram and that spirometry is a proper tool to monitor patients with chronic pulmonary diseases rather than diagnose acute dyspnea. Chest-X ray and arterial blood gases or pulse oximetry were not used as diagnostic tools in the SESAM 2 study because these techniques are usually not provided by German general practitioners. Further diagnostics may be necessary depending on the suspected diagnosis [1,14,21,22]. In the SESAM 2 study this was the case in 14% of the patients (Table 2). The SESAM 2 study did not differentiate between new and previously known dyspnea. Data from the Transition Project revealed no relevant differences in management of new vs. previously known dyspnea except for a lower frequency of physical examination in patients with known dyspnea (Table 2) (Table 3). The results of consultation or diagnoses differed depending on whether dyspnea occured as a new or previously known complaint (Table 4).

Comparable to other reasons for encounter, most dyspnoeic patients were given prescription, incapacity certificate, doctor’s advice, and other therapies (Table 3). The doctor’s advice may be to teach methods for reducing or removing dyspnea as physical training, inspiratory muscle training and pursed-lip breathing (chronic obstructive pulmonary disease), as well as coping strategies [1,5]. The doctor´s advice did improve the situation of dyspnoeic patients significantly [23]. Hospitalisation was necessary in only about 1.1% to 5.4% of the cases. The hospitalisation rate was not much higher than for other reasons for consultation, e.g. nausea and vomiting (2.7 to 9.6% [24]) but there was a higher rate of follow-up consultations (about 85%; Table 3). This was higher than e.g. for nausea or vomiting (about 65% [24]), or pruritus (about 75%; not published). The surprisingly low hospitalisation rate and the high rate of follow-up consultations can be explained by the fact that presenting for dyspnea in general practitioners’ surgeries is regularly caused by infections of the airways or exacerbation of known chronic diseases that are not dangerous *per se* (Table 5). The results of the SESAM 2 study (Table 4) confirm the findings of Okkes et al [25]: Acute bronchitis or bronchiolitis are the most common cause of dyspnea. This contradicts other published results in which diseases such as chronic obstructive pulmonary disease, asthma and chronic heart failure, which occur more frequently as reasons for chronic dyspnea and serious acute disorders like pneumonia, pulmonary embolism or pneumothorax are discussed in detail [20,22,26,27]. In children, asthma, pulmonary infection and obstruction of the upper airways were assumed to be the most frequent causes of dyspnea [3,25]. This is supported in part by Ponka et al [20] who found bronchitis at the second rank to asthma in dyspnoeic patients younger than 45 years. In patients older than 45 years, bronchitis was the third most frequent diagnosis (after chronic heart failure and chronic obstructive pulmonary disease). In a day-to-day primary care setting, dyspnea may result from other conditions. Pedersen et al [15] found no other reason for dyspnea in 16% of overweight dyspnoeic patients. Obesity was the final result of encounter in only one of 93 cases in the SESAM 2 study. Functional dyspnea is a common phenomenon [1,5,20,28]. It may be an accompanying symptom of depressions, panic attacks [28,29], hyperventilation [20,25], or pseudoangina [30]. Jolly reported that 10% of the dyspnoeic patients had psychiatric disorders whereby 79% had either a cardiovascular or a respiratory disease [31]. This is in accordance to our findings: 84.94% of the results of consultations (”diagnoses“) made arise from the International Classification of Diseases chapters that cover cardiovascular or pulmonary diseases. In the SESAM 2 study, somatoform disorders were diagnosed to be the reason for dyspnea in one case. The prevalence of a depressive episode in dyspnoeic patients was three out of 93. Diagnoses such as acute stress or anxiety disorder are also found In the Transition Project but they were rare. This is in accordance with data of others who found mental disorders as the cause of dyspnea in 3%, organic disorders in 24% of cases and stated the cause of dyspnea as uncertain in 73% [28] of the cases. The data from both the SESAM 2 study and the Transition project indicate that a specific result of encounter or diagnosis was chosen in most of the cases: Dyspnea as the result of encounter *per se* was relatively rare (4.3 and 14.1% respectively) (Table 4) (Table 5). In contrast to the Transition Project, the SESAM 2 study did not find respiratory malignancies, Hodgkin’s lymphomas, or malignancies of the breast to be causes of dyspnea. This is an effect of the different sample size between the SESAM 2 study and Transition Project (about 9,000 versus 150,000 patients). When considering dyspnea as a reason for consultation, it is essential to keep life-threatening diseasesin mind: Some underlying causes with potentially fatal outcomes have to be taken seriously [5]. However such diagnoses were either never (e.g. epiglottitis, pulmonary embolism) or only rarely (acute myocardial infarction, cardiac arrhythmia, and pneumonia) found in our study and in the Transition Project. The percentage of hospitalised patients was low in both the SESAM 2 study and the Transition Project (Table 3). This supports the thesis that acute dangerous courses of dyspnea do not regularly occur during the consultation hours. Neither the SESAM 2 study nor the Transition Project included out-of-hours and emergency services or home visits in its data.

Strengths of the recent investigation:

1. data from a day-to-day primary care setting;
2. cross-sectional are more representative;
3. estimating total morbidity avoids attention bias.

Weaknesses of the recent investigation:

1. regional character of the investigations;
2. SESAM 2: estimation of all patients was impossible;
3. SESAM 2-data do not represent episodes of care.
